# Vaginal injuries after consensual sexual intercourse — a survey among office-based gynecologists in Hamburg, Germany

**DOI:** 10.1007/s12024-022-00488-z

**Published:** 2022-06-18

**Authors:** L. Lohner, L. Nigbur, C. Klasen, I. Witzel, J. Garland, B. Ondruschka, S. Anders

**Affiliations:** 1 Institute of Legal Medicine, University Medical Center Hamburg-Eppendorf, Butenfeld 34, 22529 Hamburg, Germany; 2 Department of Gynecology, University Medical Center Hamburg-Eppendorf, Hamburg, Germany; 3 Forensic and Scientific Services, Coopers Plains, Brisbane, Australia

**Keywords:** Sexual intercourse, Vaginal injuries, Office-based gynecologists, Genital injuries, Forensic assessment practice

## Abstract

Studies on the occurrence of injuries following consensual sexual intercourse (CSI) among patients treated by office-based gynecologists are lacking. This survey aimed to assess the presence and medical relevance of vaginal injuries after CSI in gynecological office-based practice, associated risk factors, and their significance for forensic medical assessment practice. All office-based gynecologists in Hamburg, Germany (*n* = 316), were asked to fill in a one-page questionnaire via a fax survey. The questionnaire covered various aspects such as having observed CSI-related injuries, injury severity, risk factors, and concomitant factors (bleeding, need for surgical care, hospitalization). Response rate was 43.2% (*n* = 115). Overall, 83.5% of office-based gynecologists reported having observed vaginal injuries after CSI at least once and 59.1% repeatedly. Regarding maximum injury severity, 52.1% observed mucosal erosions, 32.3% mucosa penetrating injuries, and 14.6% injuries penetrating the vagina. Having observed bleeding was reported by 56.3%, 28.1% had to perform surgical suture care, and hospital admission was initiated by 20.8%. Menopause (37.5%), use of objects (19.8%), alcohol, and/or drug use (16.7%) were reported as the most frequently observed associated risk factors. Vaginal injuries after CSI have been observed by the majority of office-based gynecologists in Hamburg involving a wide spectrum of severity, including the necessity of surgical care and hospital admission. Complementing published work in clinical and emergency medicine, these findings are highly relevant to the forensic evaluation of injuries in an allegation of sexual assault, as the severity of a vaginal injury in this setting does not necessarily support a conclusion on the issue of consent.

## Introduction

Genital injuries play an increasingly important role in forensic assessment practice. Furthermore, in gynecological and forensic practice, the recognition of medical risk factors for such injuries is particularly important, as vaginal injuries can lead to life-threatening bleeding and infections [1–3]. Fatal air embolism has also been reported in single cases when vaginal injuries occur during sexual intercourse [4–6]. Vaginal injuries are described after both consensual and non-consensual sexual intercourse (NCSI), although to our knowledge, there have been significantly more studies on genital injuries after sexual assault so far [7–20]. Studies on vaginal injuries after consensual sexual intercourse (CSI) are rare and mainly limited to frequency, location of the injury, and the influence of skin color [21–28]. To date, only a few studies have evaluated possible risk factors for such vaginal injuries, and these studies were mainly carried out in medical centers and emergency departments.

Documentation of anogenital injuries following sexual assault is essential and a core area forensic examination [29]. However, the incidence of genital injuries after CSI is difficult to record as this entity is not typically investigated by physicians in general nor by forensic doctors specifically. On the one hand, these injuries are often small bleedings after superficial mucosal tears. Since minor mucosal defects have been shown to heal quickly, usually within 72 h [17, 30], the number of unreported injuries after CSI might remain high, especially given that physical intervention and documentation are not necessary in most of these cases from the viewpoint of both physicians and patients. On the other hand, many women may not present themselves to a gynecologist in the case of such an injury, for possible sociological reasons such as shame or embarrassment. Although vaginal injuries have been described more frequently after sexual assault [17], they nevertheless also occur during CSI [30]. So far, to our knowledge, studies on how often office-based gynecologists diagnose and treat vaginal injuries after sexual intercourse are scant. The presented survey aimed to assess (i) the medical relevance, occurrence, and severity of vaginal injuries after CSI of women who presented in gynecological practices in Hamburg, Germany; (ii) the evaluation of concomitant risk factors; and (iii) the significance for forensic medical assessment practice.

## Patients and method

**Fig. 1:**
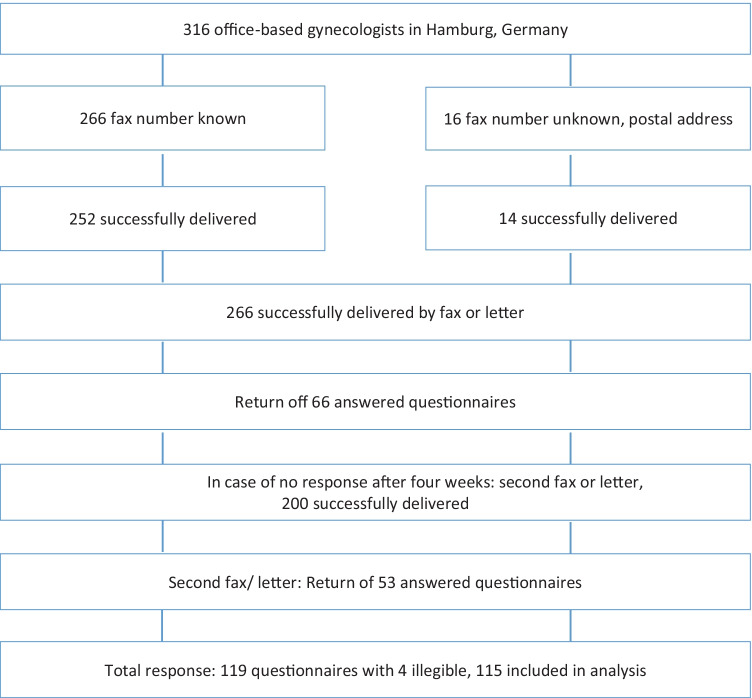
Study design

A retrospective survey among all office-based gynecologists in Hamburg, Germany, was conducted from October to November 2018. For the data collection, all gynecologists in private practice registered with the Hamburg Medical Association in October 2018 and included in a generally accessible online address list [31] were included for this survey. This involved 316 practicing gynecologists in the Hamburg urban area, with some working in joint practice. The gynecologists were contacted by fax (Simple communication GmbH, Braunschweig, Germany; www.simple-fax.de) or, if a fax number was not available, by mail using a one-page questionnaire with a covering letter. Physicians from whom no response was received after 4 weeks were contacted a second time by fax or letter (Fig. 1). A total of 266 questionnaires were successfully delivered (84.2%).

The questionnaire covered the following aspects: occurrence, risk factors, classification and localization of injuries, active bleeding, the necessity of treatment, and the necessity of hospital admission. The questionnaire was limited to one page to facilitate completion and increase the response rate, and individual items were designed to be quick to answer and without relevant effort, e.g., searching patient files. In total, the questionnaire for the office-based gynecologists contained six questions and the possibility to report back comments. Since the gynecologists were interviewed retrospectively and based only on their memories, individual questions were intentionally not overly detailed. The full questionnaire is shown in Table 1.

**Table 1:** Questionnaire and results of the survey among office-based gynecologists in Hamburg, Germany

**Questions**	**Answers by office-based gynecologists (*n*** **= 115)**
Since you have been practicing medicine in private practice, have you ever seen vaginal injuries after consensual intercourse?	No, never before: 16.5% Yes, once: 24.4% Yes, several times: 59.1%
If yes, what was the maximum depth extent you have seen in such an injury?	Mucosal erosion: 52.1% Mucosa penetrating injury: 32.3% Injuries penetrating the vagina: 14.6% Not specified: 1%
Have you ever experienced active bleeding with such an injury?	Yes: 56.3% No: 43.7%
Have you ever had to perform suture care for such an injury?	Yes: 28.1% No: 71.9%
Have you ever had to arrange for hospitalization for such an injury?	Yes: 20.8% No: 79.2%
Do you recall if any of the following risk factors were present in these cases? (Multiple answers allowed)	Menopause: 37.5% Use of objects during sexual intercourse: 19.8% Use of alcohol or drugs: 16.7% Previous obstetric surgery: 8.3% Pregnancy: 3.1% Previous injuries: 2.1%

### Design of the questionnaire

The questionnaire included a question on the occurrence of CSI-related injuries seen in gynecological office-based practice. The physicians were asked to indicate whether they had seen vaginal injuries after CSI at least once or more than once. The following questions were to be answered only if injuries had already been seen. The second question was about the maximum depth of the injuries. The gynecologists could choose between the following options: mucosal erosion (e.g., superficial erosion of the mucosa), mucosa penetrating injury (e.g., deeper injury to the tunica muscularis), or injury penetrating the vaginal wall (e.g., deepest injury to the tunica adventitia). The following three questions are related to the occurrence of active bleeding and the necessity of suture treatment or hospital admissions (binary response scheme; yes/no). In the last question, various risk factors could be marked that were present in the patients with injuries, such as pregnancy, menopause, previous obstetric surgery, use of alcohol or drugs, previous vaginal injuries due to sexual intercourse, and use of objects during sexual intercourse (multiple answers possible). In addition, there was the possibility to name further risk factors not mentioned in the questionnaire; 14 physicians made use of this possibility. An optional free text field was not used in any case.

Data were analyzed descriptively using the statistical software *IBM® SPSS® Statistics Version 24.0 (IBM, Armonk, New York, USA)*. The study protocol was approved by the Ethics Committee of the Hamburg Medical Association (Application Number WF-004/18).

## Results

The full questionnaire with results is shown in Table 1. A total of 266 gynecologists, some of them in joint practices, could be contacted by fax survey or by mail, 119 questionnaires were returned, and four of which were illegible. The evaluable response rate was thus 43.2% (*n* = 115/266).

### Occurrence of injuries

Overall, 83.5% (*n* = 96) of the responding office-based gynecologists stated that they had seen vaginal injuries following CSI at least once in their practice, with 29.2% (*n* = 28/96) reporting that they had seen such cases once and 70.8% (*n* = 68/96) at several times. Regarding the type and maximum severity of the injury, 52.1% (*n* = 50) reported mucosal erosions, 32.3% (*n* = 31) mucosa penetrating injuries, and 14.6% (*n* = 14) injuries penetrating the vaginal wall. In one questionnaire, no information was given in this regard. Active bleeding was observed by 56.3% (*n* = 54) of the respondents at least in a single case, and 28.1% (*n* = 27) reported that they experienced cases that needed suture treatment. Hospital admission was initiated by 20.8% (*n* = 20) of respondents in cases of vaginal injuries following CSI.

### Bleeding injuries, suture care, and admission rate

The number of physicians who reported observing concomitant bleeding after CSI injuries showed a dependence on the maximum injury severity observed: When mucosal injuries were reported as the most severe injury diagnosed (*n* = 50), 15 of these physicians reported to have observed active bleeding (30%). In the case of mucosa penetrating injuries (*n* = 31), this was 87.1% (*n* = 27), and when penetrating injuries were reported (*n* = 14), bleeding was observed in 78.6% (*n* = 11).

The need for surgical suture care due to injury after CSI was reported by physicians who diagnosed mucosal erosions (*n* = 50) in four cases (8%), by 45.2% reporting mucosa penetrating injuries (*n* = 14/31) and in 64.3% when penetrating injuries had been observed (*n* = 9/14). The need for hospitalization was only indicated in one case when mucosal erosions were reported as the most severe injury (2%; *n* = 50), but by 25.8% of physicians who reported to have observed mucosa penetrating injuries (*n* = 8/31) and by 78.6% in case of penetrating injuries (*n* = 11/14).

### Risk factors

Menopause (37.5%; *n* = 36), use of objects (19.8%; *n* = 19), and alcohol and/or drug use (16.7%; *n* = 16) were reported as the most frequently observed risk-increasing factors. Factors reported less frequently were previous obstetric surgeries (8.3%; *n* = 8), pregnancy (3.1%, *n* = 3), and previous vaginal injuries (2.1%; *n* = 2). In the free text field of the questionnaire, the following risk factors were also named: vaginal fisting (*n* = 1), psoriasis (*n* = 1), lichen sclerosus (*n* = 3), vaginal atrophy (*n* = 1), young age (*n* = 1), estrogen deficiency (*n* = 2), first sexual intercourse (*n* = 1), vaginal dryness due to taking hormonal contraception (*n* = 1), and masturbation with object (*n* = 1) and colpitis (*n* = 2). Due to the data structure, correlation of observed risk factors to individual injury severity is not possible.

## Discussion

Vaginal injuries after sexual intercourse are not only of medical relevance but also play a pivotal role for medicolegal expertise in cases of sexual assault. The opinion that vaginal injuries are only caused by NCSI has long been falsified [21, 23, 24, 26–28, 32, 33]. To date, a number of clinical studies on vaginal injuries following CSI focusing on emergency cases have been published [21, 23–28, 34–36]. However, these mainly refer to the frequency and localization of the injuries, while studies on risk factors are rare. To our best knowledge, there is no data on the relevance of these injuries in office-based gynecological practice.

In our survey, the majority of respondents (83.5%) reported that they had observed vaginal injury following CSI at least once in patients during their activity as office-based gynecologists; 59.1% even reported to have observed such several times. Although mucosal and therefore superficial erosions were the most frequently reported injuries, more severe lesions (mucosa penetrating injuries and injuries penetrating the vaginal wall) have already been observed by nearly half of the respondents. Consequently, injuries with active bleeding have been observed by more than half of the gynecologists, 28.1% had to perform suture care, and 20.8% had to arrange hospital admission in such cases. Although there is no information on individual age and case history due to the retrospective design of the survey, our data highlight (i) the occurrence of CSI-related injuries in everyday out-of-hospital cases and (ii) that CSI may result in severe vaginal injuries in single cases.

Most reports on vaginal injuries following sexual intercourse focus on injuries due to sexual assault (NCSI) in hospital-based and emergency department patients. The frequency of observed injuries has been described as 6–87% [7, 8, 10–16, 20, 37, 38]. The wide range of detection rates can be explained by the use of different examination techniques and different inclusion criteria of the patients (e.g., naked eye examinations vs. use of toluidine blue or erythematic changes vs. hematomas or abrasions) and different time spans post-NCSI in the inclusion criteria.

Studies on the frequency of CSI-related injuries included patients examined at hospitals, woman’s health care centers, and college students, with 6–73% of patients showing injuries [21, 23, 24, 26–28, 32, 33]. Again, the large span in detection rates can be explained by differences in individual study design.

At first glance, these results suggest that vaginal injury after intercourse may occur with equal frequency in cases of NCSI and CSI. However, comparison of the studies, which vary widely in terms of design, is probably not permitted, even given the few studies that have covered both types of injury. Lincoln et al. [23] described a frequency of macroscopically visible vaginal injuries after NCSI of 53.7% compared to 9.9% in cases of CSI with latency between event and examination of 72 h or less. The authors concluded that the risk of vaginal injury was statistically increased 19.5-fold by NCSI compared with CSI. A similar relationship in frequency, albeit lower frequency overall, was reported elsewhere (22.8% vs. 5.9%; *p* = 0.0007) [24]. Despite the apparent lower frequency of injuries after CSI based on these data, vaginal injuries after CSI were nonetheless found in a relevant proportion of the women studied. These findings are also reflected in the results of the present survey, as the overwhelming majority of office-based gynecologists reported having seen injuries resulting from CSI on one or more occasions during professional practice in private practice. Regarding the most common injury localizations, the fossa navicularis and posterior fourchette have been described [23], as well as the posterior fornix and lateral vaginal wall [27]. Due to the retrospective nature of data collection intentionally not reflecting single cases and case details and due to the forensic background of our study, the questionnaire reflected on the maximum severity of observed injuries instead of detailed anatomical injury location, a dimension that was not addressed in previously published studies. Although the present results do not allow conclusions to be drawn about the absolute frequency of mucosa penetrating injuries and penetrating injuries of the vagina, they do show that penetrating injuries, although less frequent than milder types of injuries, should not be regarded as isolated cases, as they have been sporadically described in the literature to date [39–42] since more than 14% of the physicians surveyed reported having already diagnosed such injuries. Memory bias must be considered, as more severe injuries are usually better remembered than nonspecific, superficial lesions. Nevertheless, observed bleeding, surgical suture care, and hospitalizations were reported more frequently along with more severe injuries.

Lack of prior sexual contact has been described as a risk factor for vaginal injury after NCSI [10], as has concurrent penetration with fingers in both NCSI and CSI [23]. Other risk factors for the occurrence of vaginal injuries have been described as menopausal estrogen deficiency and resulting mucosal vulnerability, prolonged sexual abstinence, use of objects, alcohol and drug use, male–female genital disproportion, prior pelvic radiotherapy, previous vaginal injury and obstetric surgery, vaginal spasm and stenosis, vaginal fisting, and coital positioning in a sitting position or with thigh hyperflexion [1, 11, 13, 15, 17, 25, 27, 35, 43–53]. The risk factors most frequently mentioned in the present survey were menopause, use of objects during sexual intercourse, use of alcohol and/or drugs, and previous gynecologic surgery, whereas other risk factors were rarely or sporadically mentioned. Overall, the feedback in the present study covered most of the risk factors known in the literature.

Our results suggest that vaginal injuries can occur during or after CSI, especially in the presence of the known risk constellations, with a wide range of severity. Not all of these injuries necessarily result in hospitalization; many of these injuries are also diagnosed and managed on an outpatient basis, even when acute bleeding and the need for suture care occur. Complementing published work in clinical and emergency medicine, our data show that vaginal injuries after CSI are not necessarily common but a well-known presentation in inpatient and outpatient gynecologic care. These findings are highly relevant to the forensic evaluation of injuries in an allegation of sexual assault, as the severity of a vaginal injury in this setting does not necessarily support a conclusion on the issue of consent, but may imply the amount of force applied.

Limitations of the survey are the retrospective data collection using a questionnaire with the resulting possibility of recall bias. Furthermore, due to the structure of the data collection, a case-specific assignment, conclusions on the absolute frequency of observations of CSI-associated vaginal injuries and the primary reason for the gynecological office visit as well as the time between CSI and gynecological examination are not possible to draw from the data and is beyond the scope of this manuscript. Moreover, the attribution of injuries to CSI as the stated mechanism of origin is based solely on the information provided by the women examined to the physicians participating in the study and the detection of vaginal injuries may be dependent on the examiner´s experience and active evaluation for injuries. The survey was limited to gynecology physicians. The inclusion of other physician professional groups (e.g., general medicine) could lead to different results. Due to the structure of healthcare in Germany, other professional groups were not included in the present survey. Minor injuries may be underrepresented in the survey because such injuries do not necessarily result in medical consultations.

Strengths include the high response rate to the questionnaires and the first-ever description of the role of vaginal injuries following CSI in outpatient gynecologic care in a forensic context. Future surveys should therefore collect detailed individual case data as part of a prospective and multicentric approach, capturing, for example, individual injury location and severity or case-specific risk factors, such as extent of alcohol association, type of previous gynecologic procedure, type and form of objects that may have been used, and the time between CSI and examination. Furthermore, hospitals and general and sexual health fields should be involved.

## Key points

1. Vaginal injuries after consensual sexual intercourse are of practical relevance for office-based gynecologists.
2. 83.5% of office-based gynecologists in Hamburg have observed this injury at least once in their practice.
3. Menopause, use of objects during sexual intercourse, the influence of alcohol or drugs, and previous obstetric surgery are risk-increasing factors for the occurrence of vaginal injuries.
4. Penetrating vaginal injuries might occur during consensual sexual intercourse and can lead to life-threatening bleeding.
5. The presence of vaginal injuries does not allow a conclusion on the issue of consent.
